# Genome-wide characterization of TCP family and their potential roles in abiotic stress resistance of oat (*Avena sativa* L.)

**DOI:** 10.3389/fpls.2024.1382790

**Published:** 2024-04-08

**Authors:** Jing Pan, Zeliang Ju, Xiang Ma, Lianxue Duan, Zhifeng Jia

**Affiliations:** Key Laboratory of Superior Forage Germplasm in the Qinghai-Tibetan Plateau, Qinghai Academy of Animal Husbandry and Veterinary Sciences, Qinghai University, Xining, China

**Keywords:** oat, TCP, genome-wide, expression profile, low nitrogen stress

## Abstract

The *TCP* gene family members play multiple functions in plant growth and development and were named after the first three family members found in this family, TB1 (TEOSINTE BRANCHED 1), CYCLOIDEA (CYC), and Proliferating Cell Factor 1/2 (PCF1/2). Nitrogen (N) is a crucial element for forage yield; however, over-application of N fertilizer can increase agricultural production costs and environmental stress. Therefore, the discovery of low N tolerance genes is essential for the genetic improvement of superior oat germplasm and ecological protection. Oat (*Avena sativa* L.), is one of the world’s staple grass forages, but no genome-wide analysis of *TCP* genes and their roles in low-nitrogen stress has been performed. This study identified the oat *TCP* gene family members using bioinformatics techniques. It analyzed their phylogeny, gene structure analysis, and expression patterns. The results showed that the *AsTCP* gene family includes 49 members, and most of the AsTCP-encoded proteins are neutral or acidic proteins; the phylogenetic tree classified the *AsTCP* gene family members into three subfamilies, and each subfamily has different conserved structural domains and functions. In addition, multiple cis-acting elements were detected in the promoter of the *AsTCP* genes, which were associated with abiotic stress, light response, and hormone response. The 49 *AsTCP* genes identified from oat were unevenly distributed on 18 oat chromosomes. The results of real-time quantitative polymerase chain reaction (qRT-PCR) showed that the *AsTCP* genes had different expression levels in various tissues under low nitrogen stress, which indicated that these genes (such as *AsTCP01*, *AsTCP03*, *AsTCP22*, and *AsTCP38*) played multiple roles in the growth and development of oat. In conclusion, this study analyzed the *AsTCP* gene family and their potential functions in low nitrogen stress at the genome-wide level, which lays a foundation for further analysis of the functions of *AsTCP* genes in oat and provides a theoretical basis for the exploration of excellent stress tolerance genes in oat. This study provides an essential basis for future in-depth studies of the *TCP* gene family in other oat genera and reveals new research ideas to improve gene utilization.

## 1 Introduction

Transcription factors (TF), also known as trans-acting factors, are a class of protein factors that can bind to specific DNA sequences (cis-acting elements) in the upstream promoter region of target genes through their DNA binding domain (DBD) and have essential roles in regulating plant growth and development (Li et al., 2019). The TCP protein, as a group of plant-specific transcription factors, evolved from algae and bryophytes and is named after the earliest identified members of the family: TB1 (TEOSINTE BRANCHED 1) in maize, CYCLOIDEA (CYC) in Cynoglossum, and Proliferating Cell Factor 1/2 (PCF1/2) in rice (Cubas et al., 1999). All members of the TCP protein contain a highly conserved basic helix-loop-helix (bHLH) structure consisting of 59 amino acid residues and can be divided into two subclasses based on the structural differences of TCP, including Class I (TCP-P/PCF) and Class II (TCP-C/CIN, TCP-C/CYC and TB1) (Viola et al., 2023). The TCP gene family is widespread in the plant kingdom, and its family members can directly or indirectly bind to regulatory target genes, which regulate multiple processes of plant growth and development (Zhou et al., 2022). In recent years, with the completion of whole genome sequencing of various species, the identification and analysis of *TCP* family genes have been carried out one after another on some species, and some related studies on the functions of the *TCP* gene family have been done (Cubas et al., 1999). *ZmTCP42* in maize (*Zea mays*) regulates some ABA or drought stress-regulated genes and is an essential positive regulator of drought tolerance (Ding et al., 2019). *MaPCF10* and *MaPCF13* of banana (*Musa acuminata*) may be functionally important in fruit development and ripening (Wang et al., 2022). *SmTCP40* of passion fruit (*Solanum melongena*) was stimulated by the jasmonic acid analog methyl jasmonate and is involved in its pigment signaling (Si et al., 2023). *DchTCP2* and *DchTCP13* in *Dendrobium chrysotoxum* significantly affect lateral organ development (Huang et al., 2023). Negative regulation of the growth of *BpTCP8*, *BpTCP14*, and *BpTCP19* meristems of *Broussonetia papyrifera* (Zhao et al., 2020). *CsTCP14*, *CsTCP16*, *CsTCP18*, *CsTCP19*, and *CsTCP20* were identified as TCP transcription factors associated with drought stress response in *Citrus sinensis* (Liu D. H. et al., 2022). *OsTCP19*, identified in *Oryza sativa*, responds to drought and salt stress and is an essential node in cell signaling that cross-links stress and developmental pathways (Mukhopadhyay and Tyagi, 2015). Thus, the *TCP* family of related genes is extensively involved in plant growth and development processes and is essential in response to abiotic stress regulation.

Oat (*Avena sativa* L.), as an annual grain-feeding crop, belongs to the grass family and is grown in about 42 countries around the world due to their excellent traits such as coolness, high nutritional value, high-stress resistance, and good palatability (Singh et al., 2013). Oat germplasm is mainly grown on marginal lands to avoid competition with grain crops for arable land. As a result, oat are often exposed to unfavorable growing conditions such as high salinity, drought, extreme temperatures, and low nitrogen stress. Nitrogen, as one of the essential nutrients in plants, is widely used in current agricultural production because its application significantly promotes plant growth and increases crop yields (Liang et al., 2021). Statistics show that the total amount of nitrogen fertilizers produced globally to increase crop yields exceeds 1 million tons annually. Although the production of N fertilizers consumes only 1-2% of global energy consumption, the utilization rate of N fertilizers is less than 50% (Dobermann and Cassman, 2005). As the global population continues to increase, overuse of nitrogen fertilizers has triggered severe environmental problems such as environmental pollution and global warming. Therefore, without increasing the use of nitrogen fertilizers, improving the nitrogen utilization rate of pasture grasses and cultivating pasture grass species with high nitrogen utilization rates or low nitrogen tolerance are of great significance for the sustainable development of the pasture industry.

The publication of the whole genome information of oat provides theoretical primary data for identifying family genes and gene function-related studies at the whole genome level. In recent years, functional genomics-related studies of oats have become a hot spot. Currently, in the available analysis of transcription factors in oat, the gene families of GRAS (Pan et al., 2023), WRKY (Liu K. et al., 2022), and PYL (Mi et al., 2023) have been characterized. Many genes have been unearthed in resistance to abiotic stresses, such as salinity, drought, cold, and high temperature. Although the *TCP* gene family members were widely used for abiotic stress response in other plants, the *AsTCP* gene family has not been characterized, and its function in response to low nitrogen stress remains unclear.

In this study, we identified and analyzed the *AsTCP* gene family members at the whole genome level and systematically analyzed the physical and chemical properties, conserved motif analysis, phylogeny, chromosomal localization, covariance, and cis-acting elements of the *TCP* gene family members. Meanwhile, this study analyzed the expression patterns of *TCP* gene family members in different tissues based on the genetic validation of qRT-PCR under low nitrogen stress, which provides a theoretical basis for further in-depth research on the molecular mechanism of abiotic stress resistance of *AsTCP* genes and the mining of excellent oat genes.

## 2 Materials and methods

### 2.1 Identification and sequence analysis of *AsTCP* genes

The oat genome was obtained from the results of joint sequencing of units studied by PepsiCo and Corteva Agriscience (https://wheat.pw.usdaa.gov/jb/?data=/ggds/oat-ot3098-pepsico). Searching for TCP candidate genes from the oat genome, this study first explored and compared the structural domains in the oat genome using the HMMER software (Version 3.0, http://hmmer.org/). The TCP protein sequence of Arabidopsis was obtained from *EnsemblPlants* (http://ensemblgenomes.org/). Hidden Markov model (HMM) mapping of the structural domains of the *Arabidopsis TCP* gene family was searched based on the oat genome, and information about the structural domains of oat was extracted using the HMMER software. To demonstrate the existence of oat TCP proteins, TCP structural domains were identified online using NCBI’s Conserved Domain Database database (CDD, https://www.ncbi.nlm.nih.gov/) and Plam values to obtain oat TCP candidate sequences, and candidate sequences whose TCP structural domains were not conserved were deleted.

### 2.2 Phylogenetic analysis of *AsTCP* genes

To further explore the evolutionary relationship between *Arabidopsis* and oat, phylogenetic analyses were constructed in this study. Multiple sequence comparisons of TCP proteins from both species were performed using MEGA software (Kumar et al., 2018) (Version X, http://megasoftware.net/). We first explored the best model for tree building based on the “Parameter” coefficients in the “Model” column, and the DNA model predictions indicated that “WAG+G+F” was the best choice. Phylogenetic tree using maximum likelihood (ML) method (calibration parameter Bootstrap, repeated 1000 times). In the parameter setting, we execute the ML method (Where “Model/Method” selects “WAG with Freqs. (+F) model”; “Rates among Sites” select “Gamma Distributed (G)”; “Test of Phylogeny” select “Bootstrap method”; “Substitutions Type” select “Amino acid”; “*No of Discrete Gamma Categories*” set to 5; “*No. of Bootstrap Replications*” set to 1000; “Gaps/Missing Data Treatment” select “Partial deletion”; “*Site Coverage Cutoff (%)*” set to 95, and the rest of the parameters are set to default values), phylogenetic tree visualization using iTOL (https://itol.embl.de/).

### 2.3 Conserved motifs analysis of oat

To study the TCP characteristic structural domains in oat TCP sequences, in this study, multiple sequence comparison of AsTCP proteins was carried out using Jalview software (Version 2.10.5), with the parameters of the comparison process set to the default values, and the results of the comparison were colorized using BioEdit software (Hall et al., 2011). AsTCP conserved protein sequences were identified using the online tool MEME Suit (Bailey et al., 2009) (https://meme-suite.org/meme/tools/meme) and visualized using TBtools software. Physicochemical properties of oat TCP proteins were predicted using the ExPASy proteomics server, and their subcellular localization was predicted using Cell-PLoc online software (http://www.csbio.sjtu.edu.cn/bioinf/Cell-PLoc-2/).

### 2.4 Chromosomal location and homology analysis of *AsTCP* genes

The CDS sequences and gene sequences corresponding to all oat TCP genes were extracted from the oat genome files using TBtools, and the intron and exon structures of the *AsTCP* genes were predicted using the GSDS 2.0 (gene structure display server, http://gsds.gao-lab.org/) online web site Analysis. The position of the *AsTCP* gene corresponding to the chromosome was obtained from the GFF3 data of the oat genome (Chen H. et al., 2020). Gene localization on chromosomes was visualized using TBtools software (Chen C. et al., 2020). To explore the homology of *TCP* genes between oat and other plant species, we downloaded genome-wide data and gene annotation files from the online website EnsemblPlants (http://ensemblgenomes.org/) for Arabidopsis (*Arabidopsis thaliana*), rice (*Oryza sativa*), maize (*Zea mays*), wheat (*Triticum aestivum*), and barley (*Hordeum vulgare*) genome-wide data and gene annotation files. In this study, we analyzed the duplication events of the *AsTCP* genes using the One Step MCscanX function of TBtools software.

### 2.5 Cis-acting elements analysis of *AsTCP* genes

The cis-acting elements of the *AsTCP* gene family were predicted and analyzed using PLACE (http://www.dna.affrc.go.jp/PLACE/signalscan) online software using the bases 2000 bp upstream of the start codon of the gene family as the sequence, which was extracted by Tbtools software (Chen C. et al., 2020). The cis-acting elements of the *AsTCP* genes were visualized by the”Simple BioSequence Viewer” function of TBtools software.

### 2.6 Plant material and stress treatments

The oat variety selected in this experiment was “Jiayan No.2”, and the seeds were provided by the Qinghai Academy of Animal Science and Veterinary Medicine, Qinghai University. This experiment began on November 20, 2023, with full-grained seeds soaked in 75% ethanol for 1 minute for disinfection and then rinsed well with distilled water for germination. The oat seedlings with consistent growth were transferred to Hoagland nutrient solution (PH= 5.8) in 16 h light/8 h darkness with a light intensity of about 8000 lx. The seedlings were then grown and cultured at 28°C until the two-leaf stage, and the nutrient solution was replaced every two days. After seedlings grew to 3 weeks of age, we divided seedlings into two groups. One group was treated with Hoagland nutrient solution containing 1.25 mM NH_4_NO_3_ for low nitrogen stress (LN- Low nitrogen), and the other group was incubated with Hoagland nutrient solution holding 10 mM NH_4_NO_3_ (NN-normal nitrogen) (Wang Y. et al., 2023). The low N treatment time points were 7 and 14 days, with a control group at normal N levels. The second leaf from the top of each plant and a root sample from each plant were then taken, immersed in liquid nitrogen for quick freezing, and stored in a freezer at -80°C. Three independent biological replicates were performed, each with 3-6 individual plants.

### 2.7 Expression analysis of the *AsTCP* genes by qRT-PCR

Total RNA was extracted from oat root and leaf tissues under different treatment times using the MiniBEST Plant RNA Extraction Kit (Takara, China). Genomic DNA was removed by reverse transcription using PrimeScriptTM RT reagent Kit with gDNA Eraser (Takara), and first-strand cDNA was synthesized. cDNA concentration was determined using a Multiskan FC zymography (Thermo Fisher, USA) and uniformly diluted to 50 ng·µL^-1^ for qRT-PCR reactions. Specific primers were designed by Primer software (Version 5.0). The qRT-PCR analysis was performed using a LightCycler Fast Real-Time PCR system (Roche, Switzerland) with three technical replicates. The amplification reaction system consisted of 2 µL of diluted cDNA solution, 1 µL of forward and reverse primers, 12.5 µL of SYBR Premix Ex Taq II (RR047A, Takara), and 8.5 µL of sterilized water in a total volume of 20 µL. The reaction program consisted of 40 cycles of denaturation at 95°C for 30 s, denaturation at 95°C for 5 s, and annealing at 60°C for 30 s; 95°C for 15 s, 60°C for 30 s, and 95°C for 15 s. This study used the GAPDH gene reported by Tajti et al. as an internal reference gene (Tajti et al., 2021). This inner reference gene is identical to the studied species and showed largely stable expression levels in different tissues. To ensure the accuracy of the experimental results, three replications were performed for each sample (**Table 1**). The relative expression was calculated using the 2^−(ΔΔCt)^ method (Livak and Schmittgen, 2001).

**Table 1:** Information on the TCP genes in oat.

Gene name	ID	Chromosome location	Amino Acid length	pI	Molecular weight (Da)	Subcellular Localization
*AsTCP01*	*AVESA.00001b.r3.1Dg0001753.1*	1D	301	6.55	32415.13	Nucleus
*AsTCP02*	*AVESA.00001b.r3.4Ag0002058.1*	4A	398	6.08	41255.02	Nucleus
*AsTCP03*	*AVESA.00001b.r3.1Dg0001753.2*	1D	301	6.55	32415.13	Nucleus
*AsTCP04*	*AVESA.00001b.r3.6Ag0000828.1*	6A	414	9.22	41220.44	Nucleus
*AsTCP05*	*AVESA.00001b.r3.2Cg0001376.2*	2C	421	9.21	45769.80	Nucleus
*AsTCP06*	*AVESA.00001b.r3.2Dg0002872.1*	2D	188	9.08	21002.88	Chloroplast.
*AsTCP07*	*AVESA.00001b.r3.2Cg0001376.1*	2C	521	10.11	56677.00	Nucleus
*AsTCP08*	*AVESA.00001b.r3.7Ag0000616.1*	7A	282	8.93	28770.43	Nucleus
*AsTCP09*	*AVESA.00001b.r3.1Ag0001719.1*	1A	301	6.65	32319.94	Nucleus
*AsTCP10*	*AVESA.00001b.r3.4Ag0003426.1*	4A	326	6.06	34448.94	Nucleus
*AsTCP11*	*AVESA.00001b.r3.2Ag0000686.1*	2A	430	9.21	46477.41	Nucleus
*AsTCP12*	*AVESA.00001b.r3.2Ag0000686.2*	2A	430	9.21	46477.41	Nucleus
*AsTCP13*	*AVESA.00001b.r3.2Cg0001376.4*	2C	521	10.11	56677.00	Nucleus
*AsTCP14*	*AVESA.00001b.r3.2Cg0001376.5*	2C	521	10.11	56677.00	Nucleus
*AsTCP15*	*AVESA.00001b.r3.6Cg0002756.1*	6C	403	9.22	41045.29	Nucleus
*AsTCP16*	*AVESA.00001b.r3.5Ag0001691.1*	5A	400	6.09	41347.30	Nucleus
*AsTCP17*	*AVESA.00001b.r3.Ung0000515.1*	Un	265	8.94	27310.72	Nucleus
*AsTCP18*	*AVESA.00001b.r3.4Ag0002057.1*	4A	380	5.94	39273.80	Nucleus
*AsTCP19*	*AVESA.00001b.r3.3Dg0000197.1*	3D	230	5.95	24178.98	Nucleus
*AsTCP20*	*AVESA.00001b.r3.7Cg0000189.1*	7C	398	6.06	41114.72	Nucleus
*AsTCP21*	*AVESA.00001b.r3.5Dg0000558.2*	5D	294	6.79	31484.90	Nucleus
*AsTCP22*	*AVESA.00001b.r3.5Dg0000558.3*	5D	347	8.84	37170.63	Nucleus
*AsTCP23*	*AVESA.00001b.r3.3Dg0001631.1*	3D	267	7.23	29302.74	Nucleus
*AsTCP24*	*AVESA.00001b.r3.1Ag0003249.2*	1A	300	6.55	32277.94	Nucleus
*AsTCP25*	*AVESA.00001b.r3.2Dg0003248.1*	2D	429	9.21	46404.31	Nucleus
*AsTCP26*	*AVESA.00001b.r3.5Dg0000558.1*	5D	347	8.84	37170.63	Nucleus
*AsTCP27*	*AVESA.00001b.r3.1Ag0003249.1*	1A	300	6.55	32277.94	Nucleus
*AsTCP28*	*AVESA.00001b.r3.2Dg0003248.2*	2D	429	9.21	46404.31	Nucleus
*AsTCP29*	*AVESA.00001b.r3.4Dg0002415.5*	4D	402	10.73	41494.26	Nucleus
*AsTCP30*	*AVESA.00001b.r3.6Ag0003090.1*	6A	137	11.03	14458.36	Nucleus
*AsTCP31*	*AVESA.00001b.r3.4Dg0002415.3*	4D	403	10.73	41595.36	Nucleus
*AsTCP32*	*AVESA.00001b.r3.4Dg0002415.4*	4D	403	10.73	41595.36	Nucleus
*AsTCP33*	*AVESA.00001b.r3.5Ag0000973.1*	5A	345	8.83	36802.23	Nucleus
*AsTCP34*	*AVESA.00001b.r3.5Dg0001267.1*	5D	400	6.00	41290.20	Nucleus
*AsTCP35*	*AVESA.00001b.r3.4Dg0002415.1*	4D	418	10.73	43158.24	Nucleus
*AsTCP36*	*AVESA.00001b.r3.4Dg0002415.2*	4D	413	10.73	42628.64	Nucleus
*AsTCP37*	*AVESA.00001b.r3.6Dg0000536.1*	6D	405	9.38	40743.01	Nucleus
*AsTCP38*	*AVESA.00001b.r3.6Dg0000536.2*	6D	405	9.38	40743.01	Nucleus
*AsTCP39*	*AVESA.00001b.r3.4Dg0002975.1*	4D	407	5.79	43341.43	Nucleus
*AsTCP40*	*AVESA.00001b.r3.7Cg0001665.1*	7C	378	7.43	39456.56	Nucleus
*AsTCP41*	*AVESA.00001b.r3.5Ag0002989.1*	5A	154	10.26	16350.52	Nucleus
*AsTCP42*	*AVESA.00001b.r3.2Dg0002872.2*	2D	187	8.78	20762.77	Chloroplast. Nucleus.
*AsTCP43*	*AVESA.00001b.r3.4Dg0003552.1*	4D	325	6.19	34394.94	Nucleus
*AsTCP44*	*AVESA.00001b.r3.3Cg0002325.1*	3C	267	7.23	29160.58	Nucleus
*AsTCP45*	*AVESA.00001b.r3.3Ag0002082.1*	3A	267	7.25	29238.70	Nucleus
*AsTCP46*	*AVESA.00001b.r3.Ung0000313.1*	Un	271	8.94	27823.32	Nucleus
*AsTCP47*	*AVESA.00001b.r3.4Dg0003552.2*	4D	325	6.19	34394.94	Nucleus
*AsTCP48*	*AVESA.00001b.r3.7Ag0001146.1*	7A	393	8.93	41438.92	Nucleus
*AsTCP49*	*AVESA.00001b.r3.4Dg0003552.3*	4D	332	6.13	35080.77	Nucleus

### 2.8 Statistical analysis

The data were organized using Microsoft Excel software, analyzed by ANOVA using SPSS 17.0 statistical software, and the least significant difference (LSD) method was used to compare the differences between groups of different data, with the level of significance set at α = 0.05. Graphing was performed using Origin Pro 2019b (Origin Lab Corporation, Inc. Northampton, Massachusetts, USA) software for graphing.

## 3 Results

### 3.1 Identification of *AsTCP* gene family members in oat

49 TCP proteins were identified in oat, named AsTCP01 to AsTCP49 based on their physical location on the chromosome (**Supplementary Table S2**), and analyzed for coding sequence length (CDS), protein molecular weight (Mw), isoelectric point (pI), and chromosomal location of the gene (Location) (**Table 1**). Among the 49 AsTCP proteins, the AsTCP41 protein was the smallest, with 154 amino acids, and the AsTCP07, AsTCP13, and AsTCP14 proteins were the largest, with 521 amino acids. The molecular weights of the proteins ranged from 14458.36 Da (AsTCP30) to 56677 Da (AsTCP07, AsTCP13, and AsTCP14), the pI values ranged from 5.79 (AsTCP39) to 10.73 (AsTCP29, AsTCP31, AsTCP32, AsTCP35, and AsTCP36) The mean pI value was 8.24, indicating that oat TCP protein was acidic.

### 3.2 Phylogenetic analysis and classification of *AsTCP* genes

**Figure 1:**
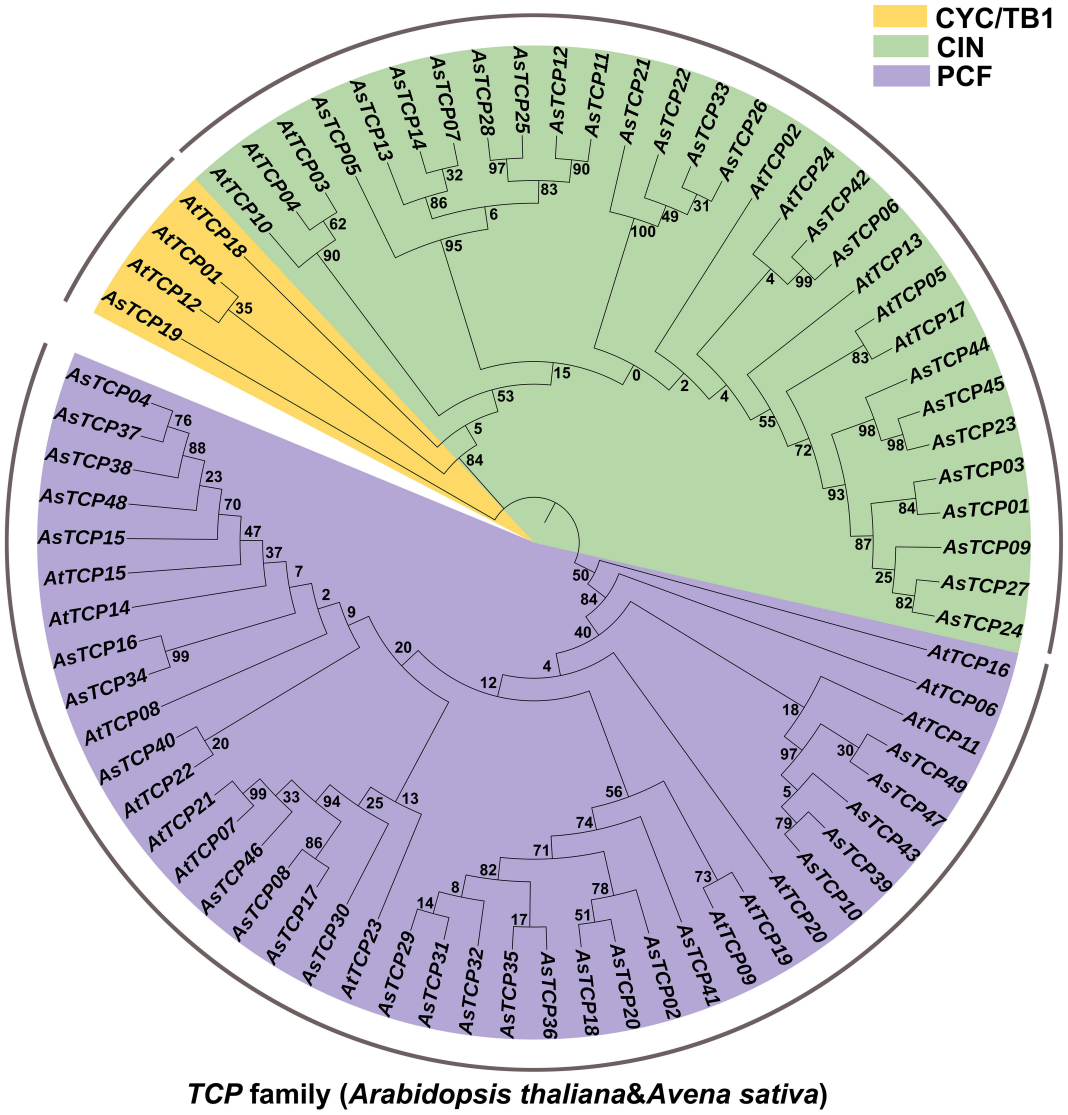
Phylogenetic tree of TCP proteins in *Avena sativa* and *Arabidopsis thaliana*. The TCP protein sequences of the two species were aligned by MEGA X with the MUS-CLE method, and the tree was built with the maximum likelihood (ML) method. The tree was further categorized into three distinct subfamilies in different colors.

To explore the phylogenetic relationships of oat TCP proteins, we constructed a phylogenetic tree based on the amino acid sequences of 49 AsTCP proteins and 24 AtTCP proteins (**Supplementary Table S3**) using the maximum likelihood (ML) method (**Figure 1**). Based on their homology with *Arabidopsis* TCP proteins, the 49 *AsTCP* genes were classified into two major branches and three subfamilies, of which the PCF subfamily included 14 *AtTCP* genes and 25 *AsTCP* genes; the CIN subfamily contained 8 *AtTCP* genes and 22 *AsTCP* genes; and the CYC/TB1 subfamily contained 3 *AtTCP* genes and 1 *AsTCP* genes (**Figure 1**). The PCF subfamily contained the most *AsTCP* genes, accounting for 51% of all *AsTCP* genes. The CYC/TB1 subfamily contained the minor *AsTCP* genes, accounting for only 2% of all *AsTCP* genes.

### 3.3 Motif and gene structure analysis

**Figure 2:**
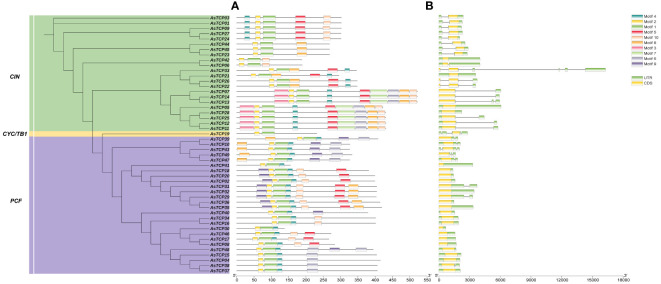
Structural analysis of *AsTCP* genes in oat. **(A)** The distribution of motifs in TCP proteins. **(B)** The exon-intron struc-ture of *TCP* gene.

The protein-conserved sequences and sequence logos of the oat TCP gene family members were analyzed using MEME online analysis software. A total of 10 conserved motifs (named motif 1~10) were identified, and more motifs were located at the C-terminal than at the N-terminal (**Figure 2A**). Most AsTCP proteins contained motif 1, motif 2, and motif 7. *AsTCP18* did not have motif 3, while *AsTCP06* and *AsTCP44* only included motifs 1 and 2. Meanwhile, comparing and analyzing the results of the phylogenetic classification of the *AsTCP* genes, we found that the types, numbers, and order of protein motifs of the members of the same subfamily were very conserved. The closely related members mostly have similar motifs: The Class I group includes 1,2 and 4, while the Class II group includes 1,2,4, and 5.

Meanwhile, we found that motif 1 and 2 appeared in all *AsTCP* genes, indicating that these motifs are strongly conserved and have important evolutionary significance for *AsTCP* gene family members. To understand the structural composition of *AsTCP* genes, the exon and intron structure maps of *AsTCP* genes were obtained by comparing the genomic DNA sequences of *AsTCP* genes (**Figure 2B**). The results showed that some *AsTCP* genes (19 genes, 39%) did not contain introns; *AsTCP05* had five introns; the number of exons in oat *TCP* genes ranged from one to two, with most of the *AsTCP* genes containing only one exon, and only *AsTCP06*, *AsTCP22*, *AsTCP26*, and *AsTCP33* contained two exons. All 49 *AsTCP* genes included an uncompiled region (UTR). From the results, it is clear that members of the same subfamily have similar gene structures.

### 3.4 Chromosomal distribution and collinearity analysis of *AsTCP* genes

**Figure 3:**
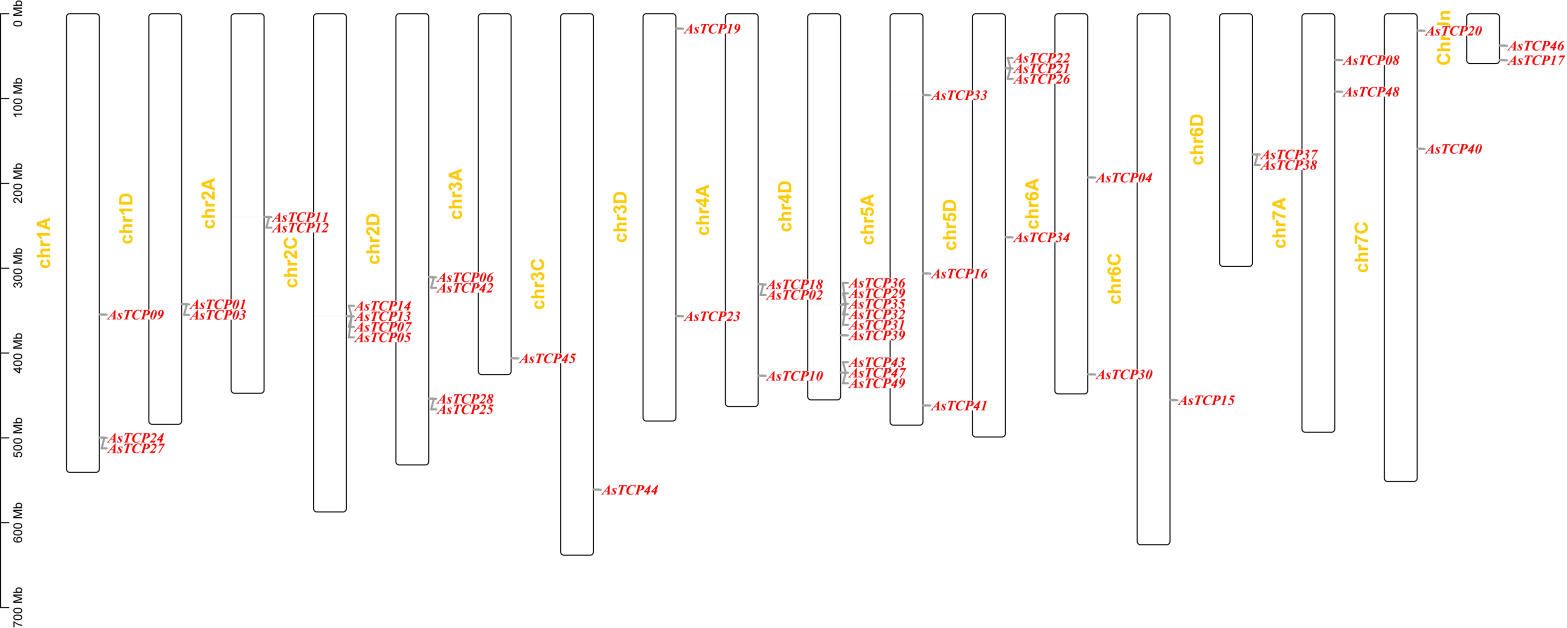
Schematic representations of the chromosomal distribution of oat *TCP* genes.

**Figure 4:**
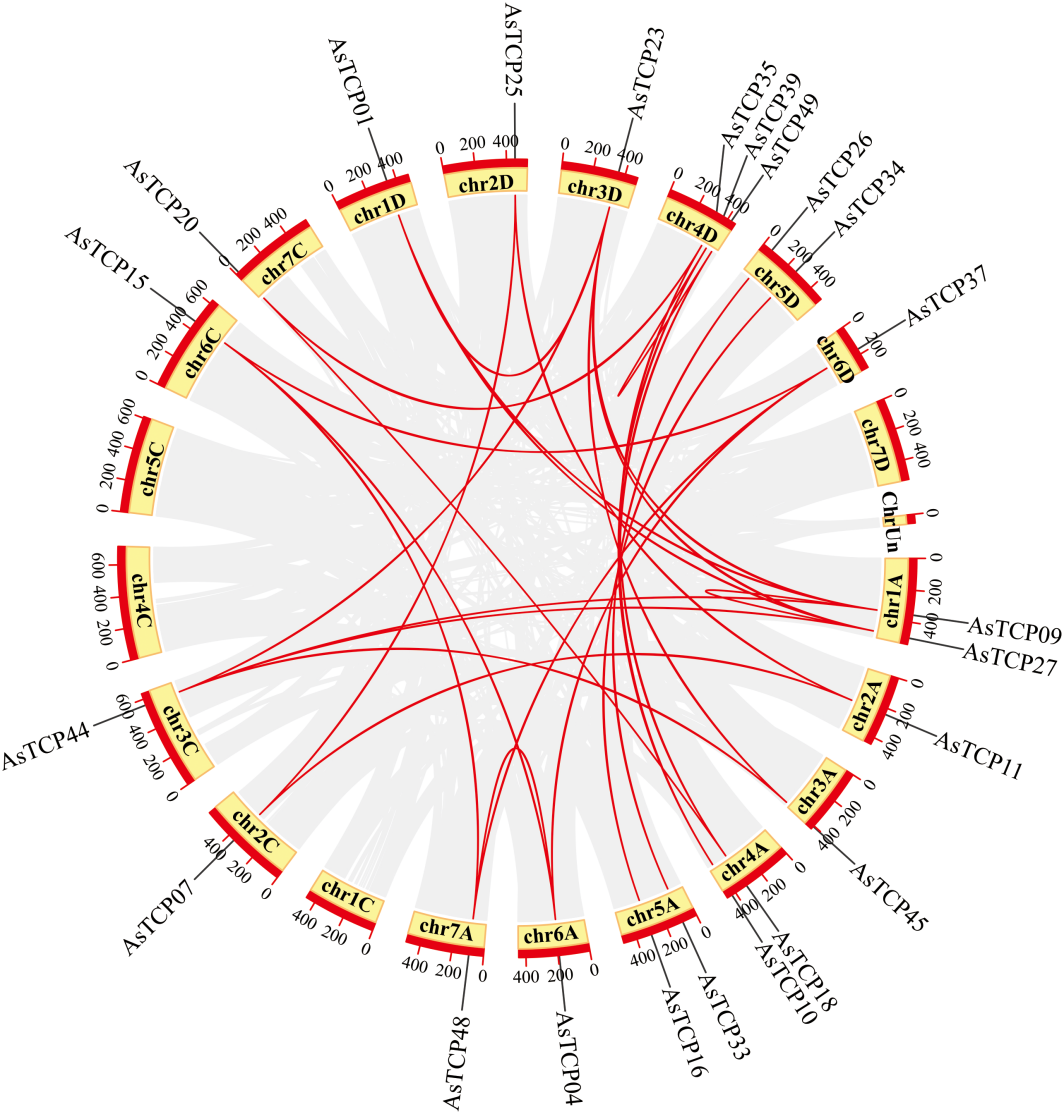
Chromosome localization of *AsTCP* duplicated genes in oat. The red lines represent the segmentally duplicated genes and the black bands represent the collinear block.

**Figure 5:**
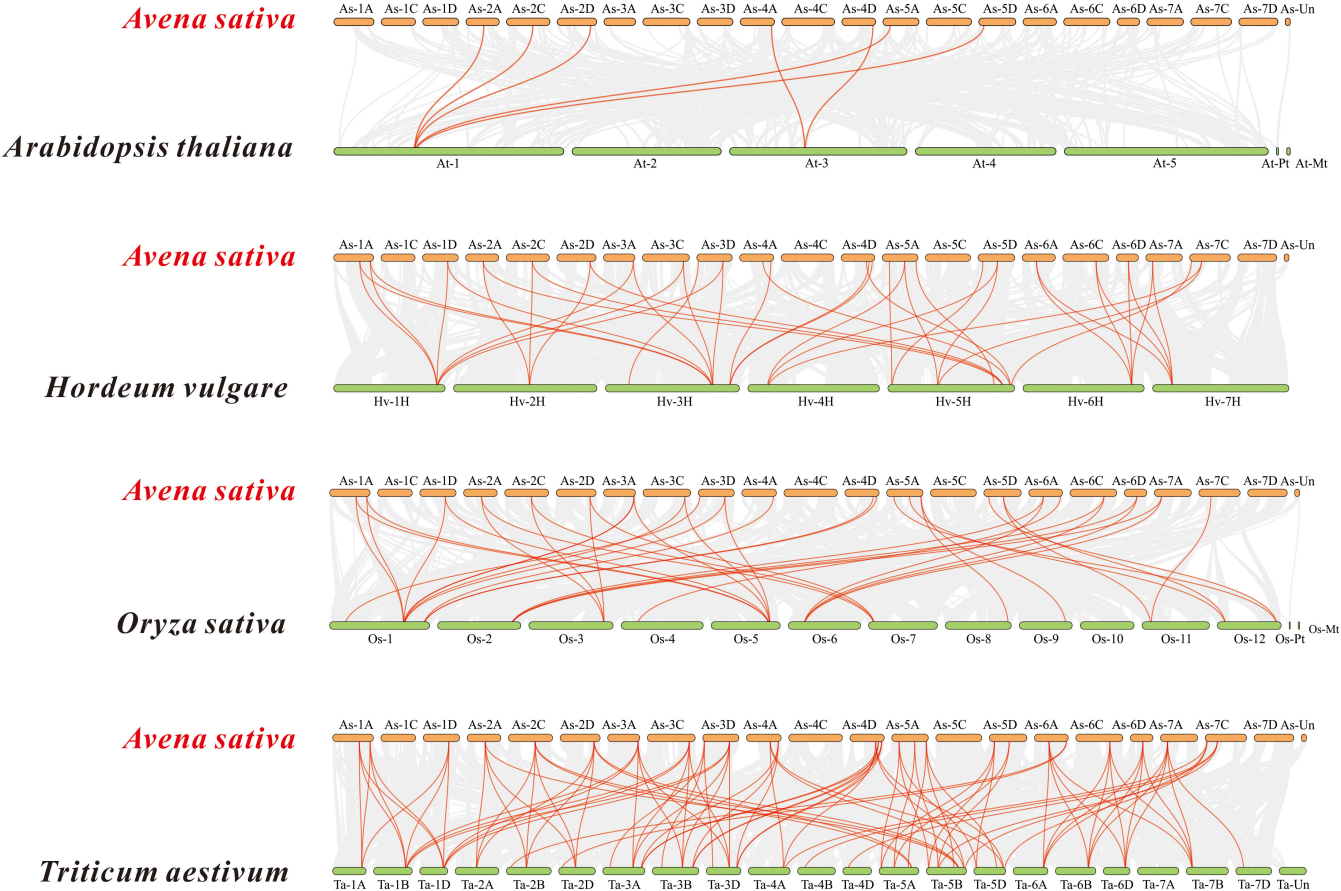
Synteny analyses of the *TCP* genes between oat and four representa-tive species. The collinear blocks within *A. sativa* and other specie genomes were displayed by the gray lines. The syntenic *TCP* gene pairs between oat and other species were highlighted with the red lines.

Based on the oat genome information, the distribution of all *AsTCP* genes on chromosomes was identified and analyzed (**Figure 3**). The results showed that 49 *AsTCP* genes were unevenly distributed on the 18 chromosomes of oat, among which nine genes were localized on chromosome 5A, which accounted for the most significant number of genes in the total number of genes, about 18.4%, followed by those on chromosome 2C (4 genes, about 8.2%), chromosome 2D (5 genes, about 8.2%) and chromosome 5D (4 genes, about 8.2%),. Only one gene was localized on chromosomes 3C, 3D, and 6C. In chromosomes, only one gene was localized (1 gene, about 2%). Tandem duplication events are essential for gene evolution and expansion. The results showed 23 regions of tandem duplication events in the oat genome (**Figure 4**, connected by red lines), indicating that these regions are hotspots for the distribution of *AsTCP* genes. The results suggest an evolutionary relationship between these *AsTCP* members, and their origins are related to chromosome duplication, implying that these genes may have similar functions. The results of collinearity analysis of *AsTCP* genes showed that genes with collinearity relationships are located within the same subfamily. To further explore the phylogenetic mechanisms of the oat TCP gene family, four representative comparative linear maps were constructed in this study to investigate the homology of *TCP* gene family members between oat and four representative species (**Figure 5**). The 4 species included 1 dicotyledon (*Arabidopsis thaliana*) and 3 monocotyledons (*Hordeum vulgare*, *Triticum aestivum*, and *Oryza sativa*). The results of covariance analysis showed that there were 7, 42, 39, and 94 homologous gene pairs between oat and *Arabidopsis thaliana*, *Hordeum vulgare*, *Oryza sativa*, and *Triticum aestivum*, respectively. Overall, the *AsTCP* genes consisted of more monocotyledonous covariant gene pairs, and the *TCP* genes were highly conserved evolutionarily between oat and *Triticum aestivum*, and the covariance analysis between oat and other species was critical for elucidating the evolution of the *TCP* genes.

### 3.5 Cis-acting element analysis of *AsTCP* genes

**Figure 6:**
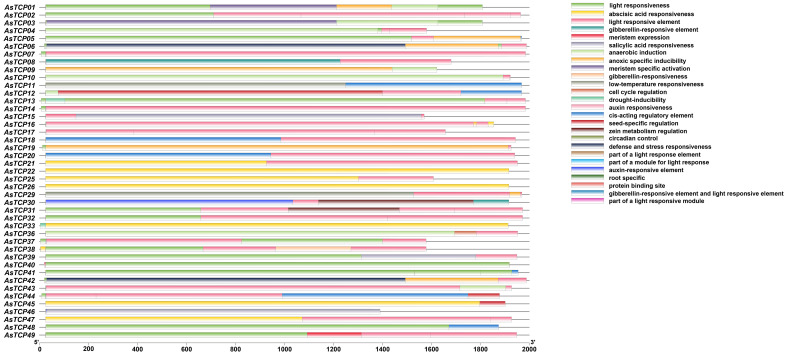
Cis-acting elements in promoters of AsTCP family members.

Cis-acting elements play essential roles in the transcriptional regulation of plant genes; they are specific binding sites involved in transcription initiation and regulation of protein binding. To predict the cis-acting elements contained in the promoter region of the *AsTCP* genes, the types of cis-elements and the potential functions of the contained elements of the *AsTCP* gene family members were analyzed using PLACE online software. The results showed (**Figure 6**, **Supplementary Table S4**) that the promoter regions of *AsTCP* gene family members contained a large number of cis-acting regulatory elements related to plant growth and development, hormone responses, and resistance, including light-responsive element (Sp1), abscisic acid-responsive element (ABRE), gibberellin-responsive element (TATC-box), low-temperature-stress-responsive element (LRE), and drought stress-responsive element (DRE/CRT) cis-acting elements. Different members of the *AsTCP* gene family contain similar cis-acting elements in their promoter regions regarding type and number. However, they also have some differences. For example, there are most *AsTCP* genes contain light-responsive elements (43 genes) and abscisic acid-responsive elements (41 genes), and some *AsTCP* genes contain drought-responsive elements (18 genes) and low-temperature stress-responsive elements (11 genes). The results showed that *AsTCP19* of the CYC/TB1 subfamily contained a higher number of cis-acting elements, and the CIN subfamily’s *AsTCP22* and *AsTCP25* genes of the CIN subfamily contained fewer cis-acting elements. The cis-acting element analyses indicated that *AsTCP* genes play critical regulatory roles against various abiotic stresses in plants.

### 3.6 Expression profiles of *AsTCP* family members under low nitrogen stress

**Figure 7:**
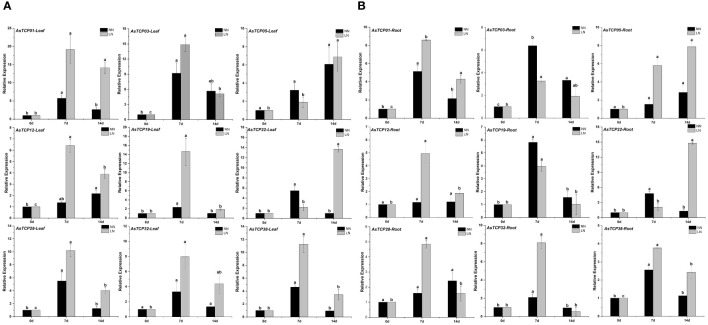
**(A)** The expression patterns of 9 *AsTCP* genes under low nitrogen stress in leaf were analyzed by real-time quantitative RT-PCR. **(B)** The expression patterns of 9 *AsTCP* genes under low nitrogen stress in root were analyzed by real-time quantitative RT-PCR. The relative expression levels which were normalized to GAPDH were determined by the comparative CT method ^(2−ΔΔCT)^ Three biological replicates were conducted for each experiment. This experiment uses the significant difference letter marking method, all the averages in order from large to small, and then in the largest average marked with the letter a, encountered with the difference between the average of the significant difference, marked with the letter b, and so on, where there is a the same marking of the letter that is, the difference is not significant, and where there is a different marking of the letter that is, the difference is significant.

To further investigate the expression levels of *AsTCP* genes in different tissues under low-nitrogen environmental conditions and to elucidate the exact functions of *AsTCP* genes, we validated 9 *AsTCP* genes among the identified *AsTCP* genes in qRT-PCR experiments, and the genes selected covered each *AsTCP* gene subfamily (**Figure 7**). Gene primers for qRT-PCR experiments were shown in **Supplementary Table S1**. According to the results of qRT-PCR experiments, some *AsTCP* genes were significantly expressed in both leaf and root tissues. The results showed differences in the expression patterns of *AsTCP* genes in different subfamilies, and the expression patterns of *AsTCP* genes in the same subfamily were similar. Under low-nitrogen environmental conditions, *AsTCP32* and *AsTCP38*, members of the PCF subfamily, reached the highest level of expression in leaf and root tissues on the seventh day of stress, followed by a gradual decrease in expression. In addition, we observed different expression patterns in members of the same subfamily. For example, in the CIN subfamily, the expression of *AsTCP03* tended to be up-regulated from 0 days to 7 days and declined at 14 days; conversely, the expression of *AsTCP05* was consistently up-regulated from 0 days to 14 days. qRT-PCR results indicated differences in the expression of some *AsTCP* genes in leaf and root tissues. For example, the expression of the *AsTCP01* gene in the CIN subfamily was higher in leaf tissues than in root tissues. In summary, by analyzing the expression patterns of different *AsTCP* genes under low-nitrogen stress, we can select some representative *AsTCP* genes to further study their importance in resisting low-nitrogen stress.

## 4 Discussion

Recently, *TCP* gene family members have begun to be identified in different plant species with the publication of genome sequencing data from other plant species. In forages, the identification of *TCP* gene families in *Medicago sativa* (Zhang et al., 2023) and *Dactylis glomerata* (Wang C. et al., 2023) has also been completed. Oat is an annual crop in the grass family, with the advantages of stress resistance and high nutritional value. It is widely used in the grass and pasture industry (Fu and Yang, 2017). However, there are no reports on *AsTCP* gene family members at the genome-wide level. Therefore, the *AsTCP* genes were systematically analyzed using published oat genome-wide information and bioinformatics techniques in this study.

This study identified 49 *AsTCP* gene family members from the oat genome. Compared with plants such as *Zea mays* (46 genes), *Triticum aestivum* (66 genes), *Hordeum vulgare* (20 genes), and *Cucumis sativus* (27 genes), the number of *AsTCP* gene family members was on the high side which demonstrated that the number of *TCP* genes was closely related to the genome size and varied among species. Species with similar gene sequences to the *AsTCP* genes are mainly from grasses such as wheat and rice, suggesting that the *AsTCP* gene family members have similar evolutionary characteristics and affinities with the genes of these closely related species. Based on the tree-like branching structure of TCP proteins between *Arabidopsis* and oat, we further classified these *AsTCP* genes into three subfamilies: PCF, CIN, and CYC/TB1. In this study, we found that the *AsTCP* genes were unevenly distributed across 18 chromosomes in the oat genome (**Figure 3**), and it is noteworthy that chromosome 5A contained the most *AsTCP* genes (9 genes), which are mainly from the PCF and CIN subfamilies. Many studies have been conducted to review the functions of plant *TCP* genes, revealing their diversity: PCF subfamily-related genes promote the proliferation of young internodal cells and activate seed embryo growth potential (Tatematsu et al., 2008). The CIN subfamily regulates plant leaf, root, and petal development (Palatnik et al., 2003). Therefore, some critical *AsTCP* genes may play a role in regulating plant growth and development.

Earlier findings showed that gene duplication transferred *TCP* gene family members from prokaryotes to land plants. Our study found some *AsTCP* genes (19 genes, about 39%) did not contain introns. This structural feature is similar to that found in *Camellia sinensis* (87%) (Shang et al., 2022), *Cymbidium goeringii* (57%) (Liu D. et al., 2022), and *Dactylis glomerata* (18%) (Wang C. et al., 2023). Most *AsTCP* genes do not have introns, meaning their transcription times are shorter than those containing introns. Evidence shows these intronless genes can produce more rapid protein (Ma et al., 2020). This study explains why the *AsTCP* genes can respond quickly and develop a response mechanism under abiotic stress. With the increase in gene sequencing data, there is growing evidence that the ancestors of all eukaryotes had genes containing introns but that most eukaryotes experienced intron loss as their species evolved. The high proportion of genes that have lost introns in these plants (e.g., *Avena sativa*, *Camellia sinensis*, and *Cymbidium goeringii*) suggests that an intron loss event may have occurred during their evolution. Most transcription factors perform functional regulation by modulating protein interactions and DNA binding activity. These regulations mainly depend on modifying structural domains and motifs of transcription factors (Liu et al., 1999). In this study, 10 conserved motifs were identified in oat, and most AsTCP proteins contained all the conserved motifs, and the types, numbers, and order of conserved motifs of proteins encoded by members of the same subfamily were relatively consistent. Notably, there are only 2 motifs (motif 1 and motif 2) in *AsTCP42*, suggesting that these motifs may have indispensable functions in the CIN subfamily. This study indicates that these 2 motifs may have essential functions for the CIN subfamily. When 2 or more genes are sequentially arranged within 200 kb of each other on a chromosome, we call this a tandem duplication event (Holub, 2001). Compared with other species, the results for *AsTCP* genes showed no significant correlation between chromosome length and the number of *AsTCP* genes.

To explore the evolutionary relationship between *AsTCP* gene family members and different plant species. We selected three monocotyledonous and one dicotyledonous plant species and analyzed the homology of their *TCP* genes. The results showed an excellent homology correlation between oat and wheat. Overall, monocotyledonous plants’ *TCP* gene family members showed better homology concordance than the identified *AsTCP* genes. Therefore, it is reasonable to speculate that the covariance between *TCP* genes correlates with the evolutionary divergence trends of the plant species in which they were found. The *AsTCP* genes originated from the same ancestor as the *TCP* gene in wheat. The results of phylogenetic clustering in angiosperms may be nuanced among species but are generally consistent. This study suggests a high degree of diversity and complexity of *TCP* genes in angiosperms (Zhao et al., 2018). Our phylogenetic tree is consistent with the phylogeny of TCP proteins from a wide range of Gramineae species (Chai et al., 2017). Oat and wheat are similar in the number of TCP proteins, but the genomic data for oat is much larger, indicating many repetitive DNA sequences in their genomes (Zhao et al., 2018). From the chromosome location map of oat (**Figure 4**), we can see the tandem duplication of multiple *AsTCP* genes, which suggests a high rate of gene duplication in oat.

The promoter region of the *AsTCP* gene family members contains many action elements, which can be classified into three major categories according to their mechanisms of action: light-responsive, stress-responsive, and hormone-responsive action elements. The results of cis-acting element analysis showed that there were six types of light-responsive elements in the promoter regions of *AsTCP* gene family members. However, different subfamily members’ types and numbers of elements varied greatly. This result indicates that various subfamily members respond differently under other light conditions. The analyses in this study showed that the *AsTCP* gene family promoters contain a variety of adversity stress-acting elements and hormone response-acting elements. Hormones were also shown to act as a trans-acting factor capable of binding to hormone-responsive elements on promoters to regulate the transcriptional expression of target genes (Gunes et al., 2007). Hormones are not only directly involved in the growth and development of organisms but also act as signal transducers (e.g., ABA) in response to adversities such as low temperature, high temperature, and salt stress (Mundy et al., 1990). Therefore, it is reasonable to speculate that the expression of *AsTCP* gene family members may be regulated by hormones, which in turn are involved in scavenging excessive reactive oxygen species from their cells *in vivo*, increasing their resistance to abiotic stresses.

With the publication of plant whole genome sequencing results, *TCP* genes involved in abiotic stress response have been identified from a wide range of plants, including species such as *Zingiber officinale* (Jiang et al., 2023), *Panicum virgatum* (Zheng et al., 2019), *Camellia sinensis* (Shang et al., 2022) and *Solanum melongena* (Li et al., 2022). This study investigated the expression patterns of *AsTCP* genes in different tissues under low nitrogen stress conditions. By performing qRT-PCR experiments, we analyzed the expression patterns of *AsTCP* gene family members under low-nitrogen stress conditions. The experimental results showed that all *AsTCP* gene family subfamilies have members that can respond to low nitrogen stress. The *AsTCP* gene family plays an essential regulatory role in the response of plants to abiotic stresses (Parapunova et al., 2014). With the publication of plant whole genome sequencing results, *TCP* genes involved in abiotic stress response have been identified from a wide range of plants, including species such as *Zingiber officinale* (Jiang et al., 2023), *Panicum virgatum* (Zheng et al., 2019), *Camellia sinensis* (Shang et al., 2022) and *Solanum melongena* (Li et al., 2022). This study investigated the expression patterns of *AsTCP* genes in different tissues under low nitrogen stress conditions. The *TaTCP9* identified were expressed throughout the development of young and immature spikes, and most *TaTCP* genes were expressed in multiple tissues and developmental stages. Expression in various tissues and developmental stages suggests that these *TCP* genes play an essential role in wheat development (Zhao et al., 2018), *ZmTCP42* identified from maize is a critical *TCP* gene in maize and plays a crucial role in response to drought stress (Ding et al., 2019), so in-depth studies on the members of the *AsTCP* gene family members are necessary for mining their superior traits and implementing molecular breeding. Therefore, in-depth research on the members of the *AsTCP* gene family is of great significance for mining its particular characteristics and implementing molecular breeding. To tap the *AsTCP* genes responsive under low nitrogen stress, we conducted a preliminary screening and found some *AsTCP* genes with significantly up-regulated expression under NH_4_NO_3_ treatment. The results showed that the expression levels of *AsTCP01*, *AsTCP03*, *AsTCP22*, and *AsTCP38* genes tended to increase during the gradual extension of low nitrogen stress. In leaf tissues, *AsTCP01* and *AsTCP03* showed higher expression levels after seven days of stress. On the other hand, *AsTCP22* remained at relatively high expression levels after 14 days of stress. In root tissues, the expression patterns were similar except for *AsTCP05* and *AsTCP22*, which showed the highest expression levels after 14 days of stress. The results of this study can further confirm that the *AsTCP* gene family has an essential role in response to low nitrogen stress. Therefore, a genome-wide survey of the *AsTCP* gene family will provide a necessary reference for the search for high-quality stress-resistant *AsTCP* genes, further deepen the understanding of the molecular mechanism of stress resistance in oat, and provide a theoretical basis for the development of excellent stress-resistant oat materials through genetic engineering technology.

In summary, in this study, members of the *AsTCP* gene family were identified at the genome-wide level using a bioinformatics approach, and their gene structures, conserved motifs, and evolutionary relationships were analyzed. Meanwhile, the potential regulatory mechanisms of the identified *AsTCP* genes in response to low nitrogen stress were revealed by determining the chromosomal distribution of the genes and their expression patterns in different tissues. In addition, the results of cis-acting element and gene expression analyses provide a basis for further investigation of *AsTCP* gene family members in abiotic stress response and other biological functions. However, the current study has only touched upon the preliminary characterization of *AsTCP* genes, and further functional validation studies are still needed to explore the roles of *AsTCP* genes in different biological processes in depth. The analysis of gene families relies on the quality of species genomes, and the quality of species genomic information affects the results of gene family analysis. As our research progresses, we will synthesize and analyze future updates of oat genomic information to sincerely resolve the theory of oat response to abiotic stress and lay the foundation for creating new highly resilient oat germplasm through genetic engineering technology.

## 5 Conclusion

In this study, we identified 49 *AsTCP* genes distributed unevenly on different chromosomes of the oat genome. Further categorizing these *AsTCP* genes, we classified them into three subfamilies: CIN, PCF, and CYC/TB1. We analyzed the gene structures and found that most *AsTCP* genes lacked introns, indicating their gene structures were very conserved. By analysis of conserved structural domains and motif patterns, we discovered that TCP gene family members of the same subfamily or branch have similar features, which may imply that they have identical gene functions. By qRT-PCR analysis, we confirmed that some *AsTCP* genes (*AsTCP01*, *AsTCP03*, *AsTCP22*, and *AsTCP38*) may play regulatory roles under low-nitrogen stress conditions, and these genes may become the focus of future research. In conclusion, this study comprehensively investigated the characterization of *AsTCP* genes. It provided a theoretical basis for further understanding the biological functions of *AsTCP* genes through the in-depth analysis of the screened particular *AsTCP* genes.

## Data availability statement

The original contributions presented in the study are included in the article/**Supplementary Material**. Further inquiries can be directed to the corresponding author.

## Author contributions

JP: Data curation, Writing – original draft, Writing – review & editing. ZLJ: Methodology, Software, Writing – review & editing. XM: Writing – review & editing. LD: Data curation, Writing – review & editing. ZFJ: Project administration, Supervision, Writing – review & editing.
